# Annotation and characterization of *Babesia gibsoni* apicoplast genome

**DOI:** 10.1186/s13071-020-04065-7

**Published:** 2020-04-21

**Authors:** Qin Liu, Long Yu, Fan Jiang, Muxiao Li, Xueyan Zhan, Yuan Huang, Sen Wang, Xiaoyong Du, Lan He, Junlong Zhao

**Affiliations:** 1 State Key Laboratory of Agricultural Microbiology, College of Veterinary Medicine, Huazhong Agricultural University, Wuhan, 430070 Hubei China; 2 Key Laboratory of Preventive Veterinary Medicine in Hubei Province, Wuhan, 430070 Hubei China; 3 Hubei Key Laboratory of Agricultural Bioinformatics, College of Informatics, Huazhong Agricultural University, Wuhan, 430070 Hubei China; 4 Key Laboratory of Development of Veterinary Diagnostic Products, Ministry of Agriculture of the People’s Republic of China, Wuhan, 430070 Hubei China

**Keywords:** *Babesia gibsoni*, Apicoplast genome, Sequencing, Annotation, Comparative analysis

## Abstract

**Background:**

*Babesia gibsoni* is an apicomplexan parasite transmitted by ticks, which can infect canine species and cause babesiosis. The apicoplast is an organelle associated with isoprenoids metabolism, is widely present in apicomplexan parasites, except for *Cryptosporidium*. Available data indicate that the apicoplast is essential for the survival of apicomplexan parasites.

**Methods:**

Here, the apicoplast genome of *B. gibsoni* was investigated by high-throughput genome sequencing, bioinformatics analysis, and conventional PCR.

**Results:**

The apicoplast genome of *B. gibsoni*-Wuhan strain (*B. gibsoni*-WH) consists of a 28.4 kb circular molecule, with A + T content of 86.33%, similar to that of *B. microti*. Specifically, this genome encodes genes involved in maintenance of the apicoplast DNA, transcription, translation and maturation of organellar proteins, which contains 2 subunits of ribosomal RNAs, 17 ribosomal proteins, 1 EF-Tu elongation factor (tufA), 5 DNA-dependent RNA polymerase beta subunits, 2 Clp protease chaperones, 23 tRNA genes and 5 unknown open reading frames (hypothetical proteins). Phylogenetic analysis revealed high similarity of *B. gibsoni* apicoplast genome to that of *B. orientalis* and *B. bovis*.

**Conclusions:**

To our knowledge, this is the first report of annotation and characterization of *B. gibsoni*-WH apicoplast genome. The results will facilitate the development of new anti-*Babesia* drug targets.
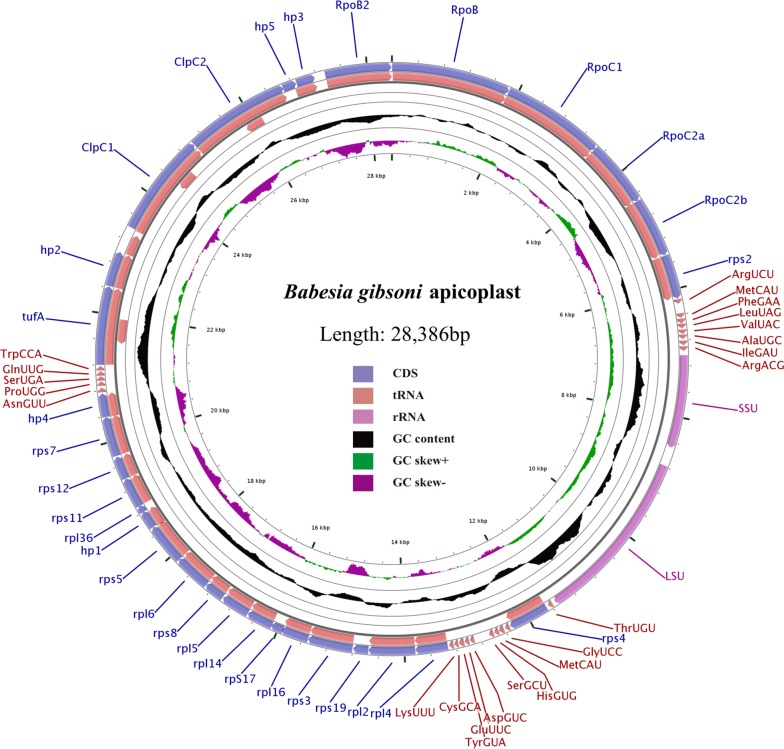

## Background

*Babesia gibsoni*, an obligate intraerythrocytic parasitic protozoan that can cause canine babesiosis [1–3], spreads widely around the world. Canine babesiosis caused by *B. gibsoni* may cause hyperacute, acute and more commonly chronic infection [3], with the clinical symptoms of fever, hemolytic anemia and even death [4, 5]. *Babesia gibsoni* is mainly transmitted by ticks but can also be transmitted by blood transfusion, dog bites and even transplacental route [6–9]. In an effort to improve the diagnosis and treatment of canine babesiosis, many research efforts have been devoted to pathogenicity, gene diversity and identification of new targets against the disease.

Apicomplexan parasites, except for *Cryptosporidium*, contain an apicoplast, which is a unique, essential organelle for the survival of these parasites [10]. The apicoplast has been reported to contain four membranes in apicomplexan parasites [11, 12] and results from a secondary endosymbiosis of eukaryotic algae, and that the red algae origin hypothesis is more likely than the green algae origin hypothesis [13, 14]. After the evolution from chloroplast into apicoplast, the genes encoding a variety of proteins, transfer RNAs (tRNAs) and ribosomal RNA (rRNA) are still present in apicomplexan parasites [15]. This endowed the apicoplast with a series of bacteria-like pathways to replicate and express its genome coupled with an anabolic capacity for producing fatty acids, iron-sulphur cluster, haem and isoprenoid precursors [16–20]. A previous study has shown that interfering the apicoplast in laboratory conditions (such as interference with isoprenoid synthesis pathways) could cause parasite death, but the addition of relevant substitutes after interference does not affect parasite growth [21]. This confirms the importance of apicoplast for parasite survival, making it a hotspot in anti-parasitic drug design.

The apicoplast genomes of *Babesia bovis*, *Babesia microti*, *Babesia orientalis* and *Toxoplasma gondii* have been sequenced and characterized [12, 22–24]. However, little information is available about the apicoplast genome of *B. gibsoni*. Therefore, this study focused on the annotation and characterization of *Babesia gibsoni*-Wuhan strain (*Babesia gibsoni*-WH) apicoplast genome.

## Methods

### Parasites and animal experiments

*Babesia gibsoni*-WH was stored in liquid nitrogen in the State Key Laboratory of Agricultural Microbiology, Huazhong Agricultural University, China.

Two one-year-old beagles with health certificates were purchased from Anlu Laboratory Animal Center and confirmed to be *Babesia-*free by microscopic examination and PCR. After injection with 5 ml of blood infected with *B. gibsoni*, the body temperature of the experimental beagles was observed daily and blood smears were prepared every second days to monitor the parasitemia. When parasitemia reached 20%, blood was collected from the experimentally infected dogs for further research as described below.

### Purification of parasites

The blood was collected from infected dogs into tubes containing EDTA-K_2_ solution, followed by storage at 4 °C for 2 h and centrifugation at 2000× *rpm* at 4 °C for 10 min. After removing the supernatant, a five-fold volume of hypotonic RBC lysis buffer (Na_3_PO_4_/EDTA, PH 7.6) was added to the pellets, followed by incubation at 37 °C for 30 min. Next, the lysate was filtered through a 2 μm membrane (Whatman, Florham Park, USA) and the collected buffer was centrifuged at 12,000×*g* at 4 °C for 10 min. Finally, the pellets were collected and stored at − 80 °C until further use.

### DNA extraction

Genomic DNA was extracted from purified parasites using the QIAamp DNA Mini Kit (Qiagen, Shanghai, China) according to the manufacturer’s instructions. After passing the quality test, the genomic DNA was used for further analysis.

### Apicoplast genome sequencing

The raw apicoplast sequence and the whole genome sequence (unpublished data) of *B. gibsoni* obtained from PacBio sequencing (Wuhan, China) were assembled using the CAP3 assembly program to obtain the raw sequence [25], which was used as reference to obtain highly conserved genes through BLAST alignment. Based on the conserved genes, primers were designed for conventional PCR amplification, followed by amplifying the sequence between two conserved genes (Additional file 1: Table S1). After several calibrations, the entire apicoplast genome was completely covered. The sequences were assembled again to obtain the final apicoplast genome sequence.

### Analysis and annotation

The apicoplast genome of *B. gibsoni* was annotated by Artemis (https://www.sanger.ac.uk/resources/software/artemis/) in combination with BLAST in NCBI (http://blast.ncbi.nlm.nih.gov/Blast.cgi). The data was screened by Open Reading Frame Finder (ORFs) (https://www.ncbi.nlm.nih.gov/orffinder/) and GenMark (http://opal.biology.gatech.edu/GeneMark/index.html). Published protozoan apicoplast genomes were used as references to conceptually translate and annotate the putative code areas. The tRNA genes were predicted by tRNAscanSE 2.0 (http://lowelab.ucsc.edu/tRNAscan-SE/) with the following parameters: source ‘‘Mito/Chloroplast’’ and search mode ‘‘default’’. The rDNA sequences (*SSU* and *LSU*) were separately obtained by comparing the counterparts of *B. bovis*, *B. microti* and *B. orientalis*. Mis-annotations in the published apicoplast genomes were re-annotated. The maps of the *B. gibsoni* apicoplast genome were produced using online CGView software (http://stothard.afns.ualberta.ca/cgview_server/). The repeatability of rRNA genes was verified using the repeatmasker server (http://www.repeatmasker.org/cgi-bin/WEBRepeatMasker). The multiple genome alignments were generated by the software Mauve (http://darlinglab.org/mauve/mauve.html). All of the above analysis results were corrected manually on a gene-by-gene basis as needed. The reference genomes used in this study included NC_011395 (*B. bovis*), KT428643.1 (*B. orientalis*), KX881914.1 (*Babesia* sp. Xinjiang), LK028575 (*B. microti*), NC_007758 (*T. parva*), X95275 (IRA) and X95276 (IRB) (*P. falciparum*), U87145 (*T. gondii*), NC_014345.1 (Alveolata sp. CCMP3155). The phylogenetic tree of the rRNA genes was constructed using MEGA 7.0 with the Maximum Likelihood method (https://www.megasoftware.net/). The functional domains and transmembrane domain were separately predicted by using the software Pfam (http://pfam.sanger.ac.uk/) [26] and TMpred (http://www.ch.embnet.org/software/TMPRED_form.html).

## Results and discussion

### Characteristics of apicoplast genome sequence of *B. gibsoni*

The available literature has shown limited genetic information of *B. gibsoni*, and the apicoplast genome sequence remains unavailable. Herein, we sequenced, assembled, annotated and characterized the apicoplast genome of *B. gibsoni*. The raw apicoplast genome sequence of *B. gibsoni* obtained from high throughput genome sequencing (data unpublished) was shown to have a circular form with a full length of 46 kb, which is bigger than that of any other reported *Babesia* apicoplast genome [22, 23, 27–29]. To further verify the sequence, we adopted a primer-walking approach to test the *B. gibsoni* genomic DNA by designing a series of specific primers based on the amplicons covering the whole apicoplast genome (Additional file 1: Table S1, Additional file 2: Figure S1). According to the principle of primer design, the sequences of conserved genes were amplified first, followed by amplifying the sequences between two conserved genes until the entire apicoplast genome was completely covered. Data analysis indicated that the assembled circular molecule has two completely repeating fragments (1–17,648 and 28,333–45,999) with a size of 17.6 kb. The apicoplast genome of *B. gibsoni* was shown to be a circular DNA of 28.4 kb with A + T content of 86.33%, which is similar to that of *B. microti* (28.7 kb, A + T% = 86%) (Table 1) [23].

**Table 1:** Characterization of *Babesia gibsoni* apicoplast genome and comparison with related species

Species	*Babesia gibsoni*	*Babesia microti*	*Babesia orientalis*	*Babesia bovis* T2Bo	*Babesia* sp. Xinjiang	*Theileria parva* Muguga	*Plasmodium falciparum* C10	*Toxoplasma gondii* RH
Size (bp)	28,386	28,657	33,200	33,351	30,729	39,579	34,682	34,996
A+T (%)	86.33	86	78.9	78.2	81	80.5	86.9	78.6
Inverted repeat	–	–	–	–	–	–	+	+
*suf*B	–	–	–	–	–	–	+	+
*rpl*23	–	–	–	–	–	–	+	–
tRNA	23	24	24	24	25	24	34	33
GenBank ID	MN481613	LK028575	KT428643.1	NC_011395	KX881914.1	NC_007758	X95275, X95276	U87145

*Note*: “Inverted repeat” indicates the information of the two genetic structures of *LSU* and *SSU*

**Fig. 1:**
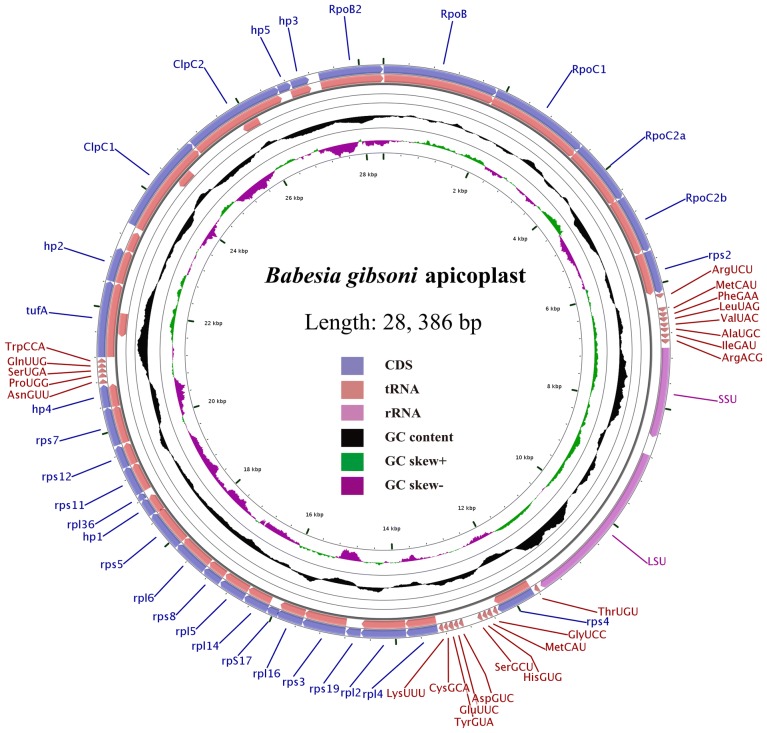
Circular map of the apicoplast genome of *Babesia gibsoni*. The map was designed using CGview. Different colors: coding sequence (CDS), tRNA, rRNA, GC content, GC skew and base coordinates. Hp1–5 represent five hypothetical protein-encoding genes found in the apicoplast genome of *B. gibsoni*

The *Babesia gibsoni* apicoplast genome contains 2 rRNAs, 23 tRNAs and 30 protein-coding genes (Table 2). Specifically, the 2 rRNA genes are composed of *SSU* and *LSU* genes. The 30 protein-coding genes comprise an EF-Tu elongation factor (*tuf*A) gene, 2 Clp protease chaperone genes (*Clp*C1, *Clp*C2), 5 DNA-dependent RNA polymerase genes (*Rpo*B, *Rpo*B2, *Rpo*C1, *Rpo*C2a, *Rpo*C2b), 17 ribosomal proteins (rpl- and rps-) and 5 hypothetical proteins (hp 1–5), with all of them in the same direction (Fig. 1, Table 2). The initiation codons are almost all AUG for the 30 CDSs (codon sequences), excluding UAU for *Rpo*B. There is an A-rich region in front of these CDS, which may play a significant role in the renewal of the ribosome population. UAA (26 of the 30 CDSs) and UGA (4 of the 30 CDSs) serve as termination codons for the CDSs of *B. gibsoni* apicoplast genome. The *sufB* gene of the protein involved in the iron-sulfur cluster assembly in the apicoplast genome of *P. falciparum*, which is absent in the apicoplast genome of *B. gibsoni* [12]. Complete annotation of the plastid genome indicated that *B. gibsoni* is more similar to *Babesia* spp. than to *Toxoplasma* or *Plasmodium* (Additional file 3: Figure S2). Additionally, almost all genes present in the apicoplast genomes of *B. orientalis* and *B. microti* were found in the apicoplast genome of *B. gibsoni*.

**Table 2:** Gene contents of the *Babesia gibsoni* apicoplast genome

Class	Gene
Ribosomal RNA	*LSU*, *SSU*
Transfer RNA^a^	Arg^UCU^Ala^UGC^Arg^ACG^Asp^GUC^Asn^GUU^Cys^GCA^Gly^UCC^Gln^UUG^Glu^UUC^His^GUG^Ile^GAU^Leu^UAG^Lys^UUU^Met^CAU^Met^CAU^Phe^GAA^Pro^UGG^Ser^GCU^Ser^UGA^Thr^UGU^Tyr^GUA^Trp^CCA^Val^UAC^
Ribosomal proteins	*rps*2, 3, 4, 5, 7, 8, 11, 12, 17, 19
	*rpl*2, 4, 5, 6, 14, 16, 36
RNA polymerase	*rpo*B, *rpo*B2, *rpo*C1, *rpo*C2a, *rpo*C2b
Other proteins	*Clp*C1, *Clp*C2, *tuf*A
Unassigned ORFs	5 ORFs (*hyp*1–5)

^a^Three-letter amino acid code and anti-codon

It has been reported that there are basically 24 tRNAs in *Babesia* spp. and more than 30 tRNAs in *Plasmodium* and *Toxoplasma* of the apicomplexan parasites (Table 1). The tRNAscan-SE analysis identified 23 tRNAs in *B. gibsoni*, which was less than that in other *Babesia* spp. Excluding one intron-containing tRNA (Leu-UAA), all the other tRNAs were the same as those in the apicoplast genomes of *B. gibsoni* and *B. orientalis* (Additional file 1: Table S2). Previous reports have shown that the apicoplast is derived from a secondary endosymbiont of eukaryotic algae, and the genomic information also indicates that the apicoplast genome conforms to the characteristics of the prokaryotic genome [10]. In prokaryotes, tRNA-fMet is an initiation tRNA. Interestingly, Met and iMet exist in both *B. microti* and *B. orientalis*, while there is only tRNA-Met in *B. gibsoni*.

Analysis revealed conspicuous similarities of rRNAs and ribosomal proteins from the apicoplast genome of *B. gibsoni* with those of other apicomplexan parasites. The apicoplast genome encodes proteins of large ribosomal subunits and small ribosomal subunits, containing 7 rpl proteins and 10 rps proteins, respectively. Genome-wide analysis of *B. gibsoni* suggests that the genes of the remaining ribosomal proteins may be transferred to the nuclear genome and then targeted to the apicoplast for function (unpublished data). This finding is also consistent with the observations in other apicomplexan parasites [30–32]. Highly conserved *16S*-like and *23S*-like rRNA genes were predicted in the apicoplast genome of *B. gibsoni*, while *5S* rRNA encoding the *rff* gene was absent in the apicoplast genomes of *B. gibsoni* and other apicomplexan parasites [22, 23, 33]. This implies that the apicoplast ribosomes of *B. gibsoni* are not regulated by *5S* rRNA, or the *rrf* gene sequence is different from that of the known *rrf* genes, or import *5S* rRNA from the cytoplasm [34, 35]. The *Chromera* chloroplast genome was reported to express the *rff* gene, while no *rrf* gene was identified in the apicoplast genomes of all apicomplexan parasites, except in the nuclear genomes [23].

In addition to the genes for tRNAs and rRNAs, the apicoplast genome *B. gibsoni* encodes genes for 2 copies of ClpC chaperone, 5 subunits of RNA polymerase, an EF-Tu elongation factor and 5 hypothetical coding sequences (hp 1–5). Except for the hypothetical coding sequences, the other genes shared a high homology in the apicoplast genomes of the apicomplexan parasite. The encoded putative proteins (hp 1–5) showed no significant homology to any of the proteins in the existing database and did not contain any identifiable functional domains. Compared with other apicomplexan parasite genomes, the results suggest the existence of hypothetical proteins with unknown functions and the different sequences in the same genomic regions. The expression of these CDS remains to be determined.

### Characteristics of gene clusters in the apicoplast genome of *B. gibsoni*

The synteny of the apicoplast genome of *B. gibsoni* with those of other apicomplexan parasites was investigated by comparing the genetic structure of four clusters in their apicoplast genomes and the chloroplast genomes of *Chromera* algae [36].

**Fig. 2:**
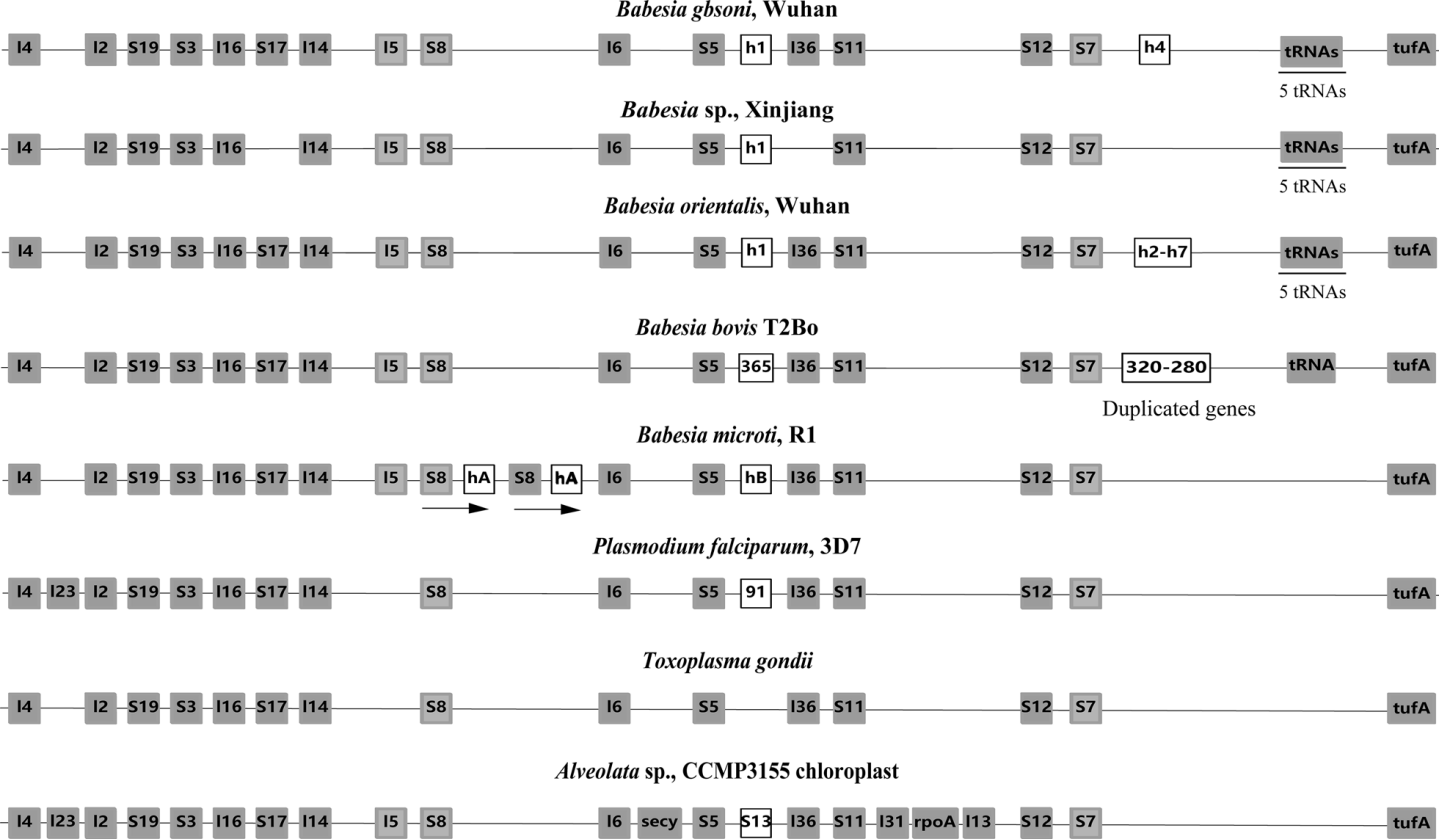
Gene structure of Cluster 1 in the apicoplast genomes of *Babesia gibsoni* (Wuhan), *Babesia* sp. Xinjiang, *B. orientalis* (Wuhan), *B. bovis* (T2Bo), *B. microti* (R1), *Plasmodium falciparum* (3D7), *Toxoplasma gondii* and the chloroplast genome of *Chromera* sp. (CCMP3155). The white box represents a unique gene of a species. The light gray box corresponds to highly divergent genes. *Abbreviations*: l, rpl (ribosomal protein large subunit); s, rps (ribosomal protein small subunit); h, hp (hypothetical protein)

Cluster 1 contains an EF-Tu elongation factor (*tuf*A) and genes encoding ribosomal proteins (Fig. 2). In this Cluster, a relatively high homology was shown in the chloroplast genome of *Chromera* algae and the apicoplast genome of apicomplexan parasites. The *rpl*23 gene is absent in the apicoplast genome of *B. gibsoni*, which is similar to the gene structure of *Babesia* sp. Xinjiang, *B. bovis* and other *Babesia* spp. The *rpl*23 gene is located between the *rpl*2 gene and the *rpl*4 gene, which was only identified in *Chromera* and *Plasmodium* species, but this gene has been lost in the apicoplast genome during evolution of apicomplexan parasites. In Cluster 1, the *rps*13 gene was only present between *rps*5 and *rpl*36 in *Chromera* sp. and absent in all apicomplexan parasites. As shown in Fig. 2, this region was replaced by a gene with unknown function, or lacked CDS at most apicomplexan parasites, like in *T. gondii*.

**Fig. 3:**
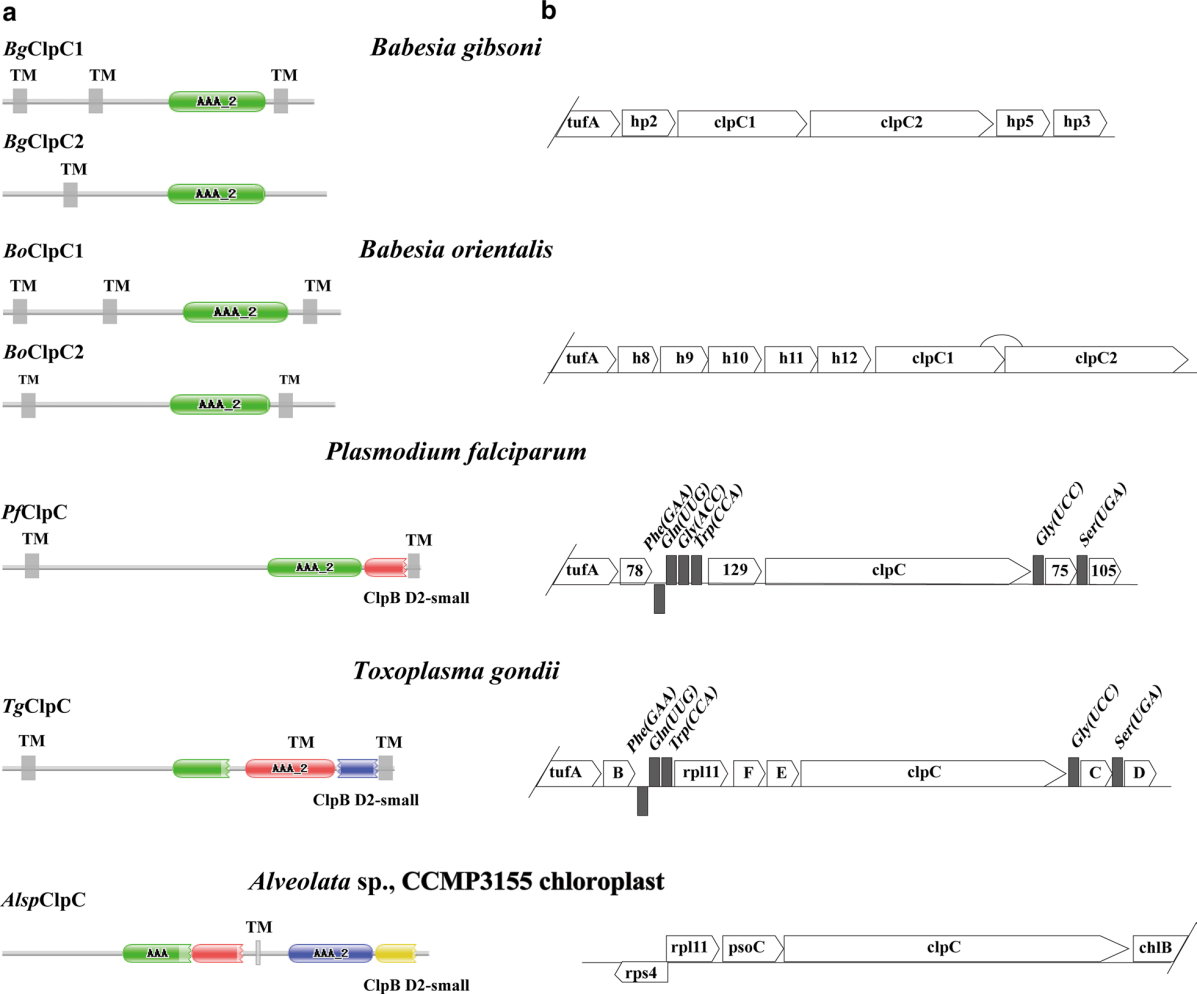
Characteristics of Cluster 2 genes and domain structure. **a** Transmembrane regions and domain structures of the *Clp*C gene forecasted by using Pfam and TMpred sequence analyzers. **b** Gene structure of Cluster 2. In *Babesia*, the conservation of *Clp*C chaperones (*Clp*C1 and *Cpl*C2) is relatively higher than that of *Toxoplasma* spp. and *Plasmodium* spp. *Abbreviations*: AAA_2, ATPase catalytic function; TM, transmembrane regions

**Fig. 4:**
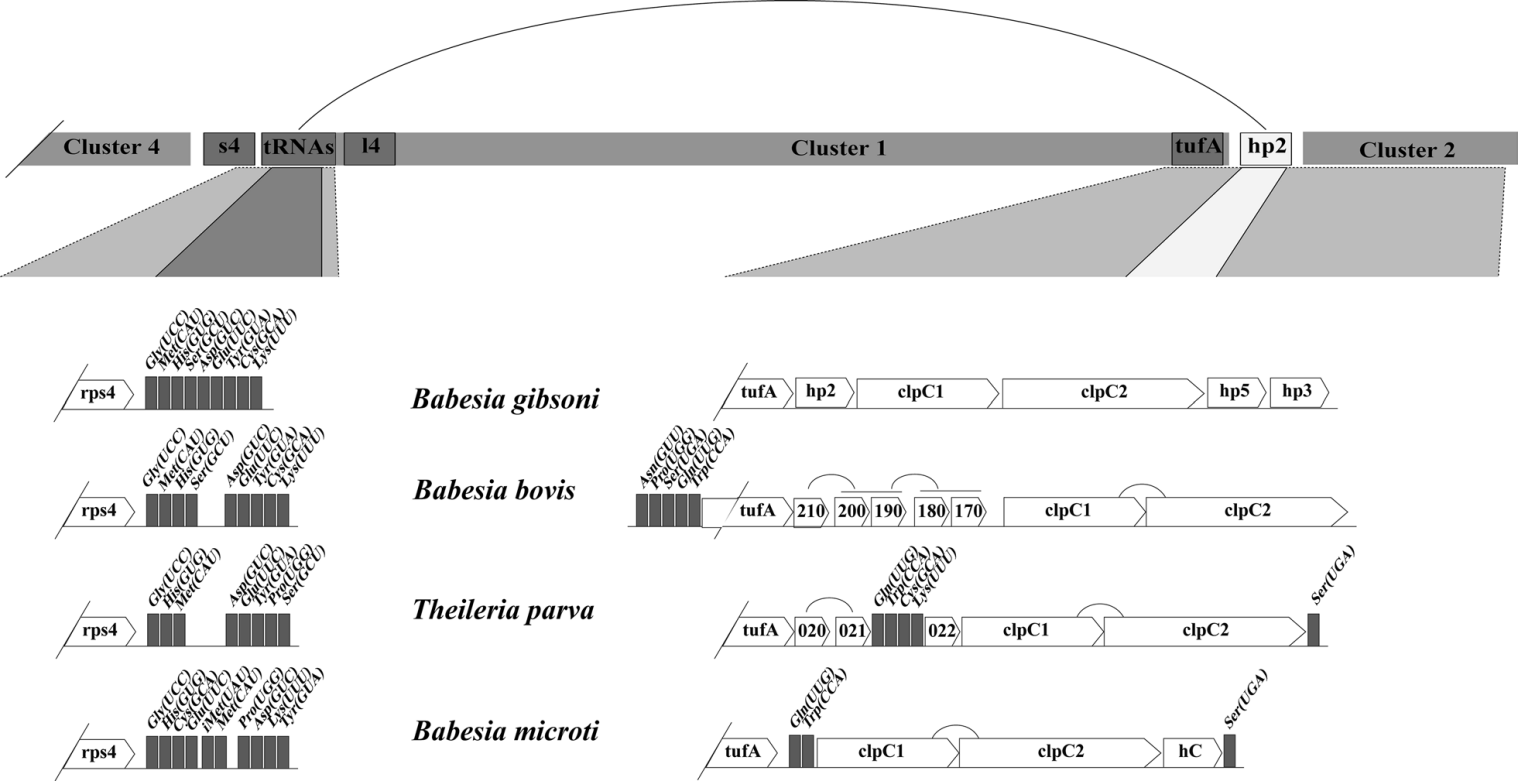
Genetic structure of the adjacent region of Cluster 1 in piroplasms. Between Cluster 1 and Cluster 4, the tRNA groups adjacent to *rps*4 were highly conserved in *Babesia* spp. A line connecting the two ends of Cluster 1 indicates possible reorganization events

Cluster 2 is mainly composed of *Clp*C chaperones. In apicomplexan parasites, the *Clp*C gene contains transmembrane domains (TM) and putative protein domains on both sides (Fig. 3). Similar to *B. microti* and *B. bovis*, the *Clp*C gene of *B. gibsoni* is also composed of two copies containing the AAA_2 ATPase domains (Additional file 4: Figure S3). Unlike *Babesia* spp., there is only one copy containing the enzyme domain for *T. gondii* and *P. falciparum*. In this cluster, the *Clp*C genes of *T. gondii* and *P. falciparum* are surrounded by tRNAs, which is not the case for *B. gibsoni* and *B. orientalis*. There is an *rp*l11 ribosomal gene in the chloroplast genome of *Chromera* sp., which was absent in the apicoplast genomes of all the other known apicomplexan parasites excluding *T. gondii* (Figs. 3, 4). This suggests that the rpl11 protein may be non-essential or contains one or more rpl11-like proteins or that this gene is encoded in the nuclear genome and then transferred to the apicoplast through the relevant pathway [30, 37].

Cluster 3 contains RNA polymerase genes (*Rpo*B, *Rpo*B2, *Rpo*C1, *Rpo*C2a, *Rpo*C2b) and the *rps*2 gene encoding the S2 ribosomal protein (Fig. 1). In algal chloroplast genomes, the alpha subunit of RNA polymerase (*Rpo*A) gene is present in Cluster 1, whereas the *Rpo*A gene is absent in the plastid genomes of *B. gibsoni* and other apicomplexan parasites, which was finally found to be encoded by the nuclear genome (Fig. 2). In the present study, 5 RNA polymerase genes were identified in *B. gibsoni*, one more than the number in *B. orientalis* or *B. microti.* However, the gene content and location of Cluster 3 are basically the same as those in *B. gibsoni*, *B. microti* and *T. parva*, indicating that it is a conserved region during evolution of Piroplasmida.

**Fig. 5:**
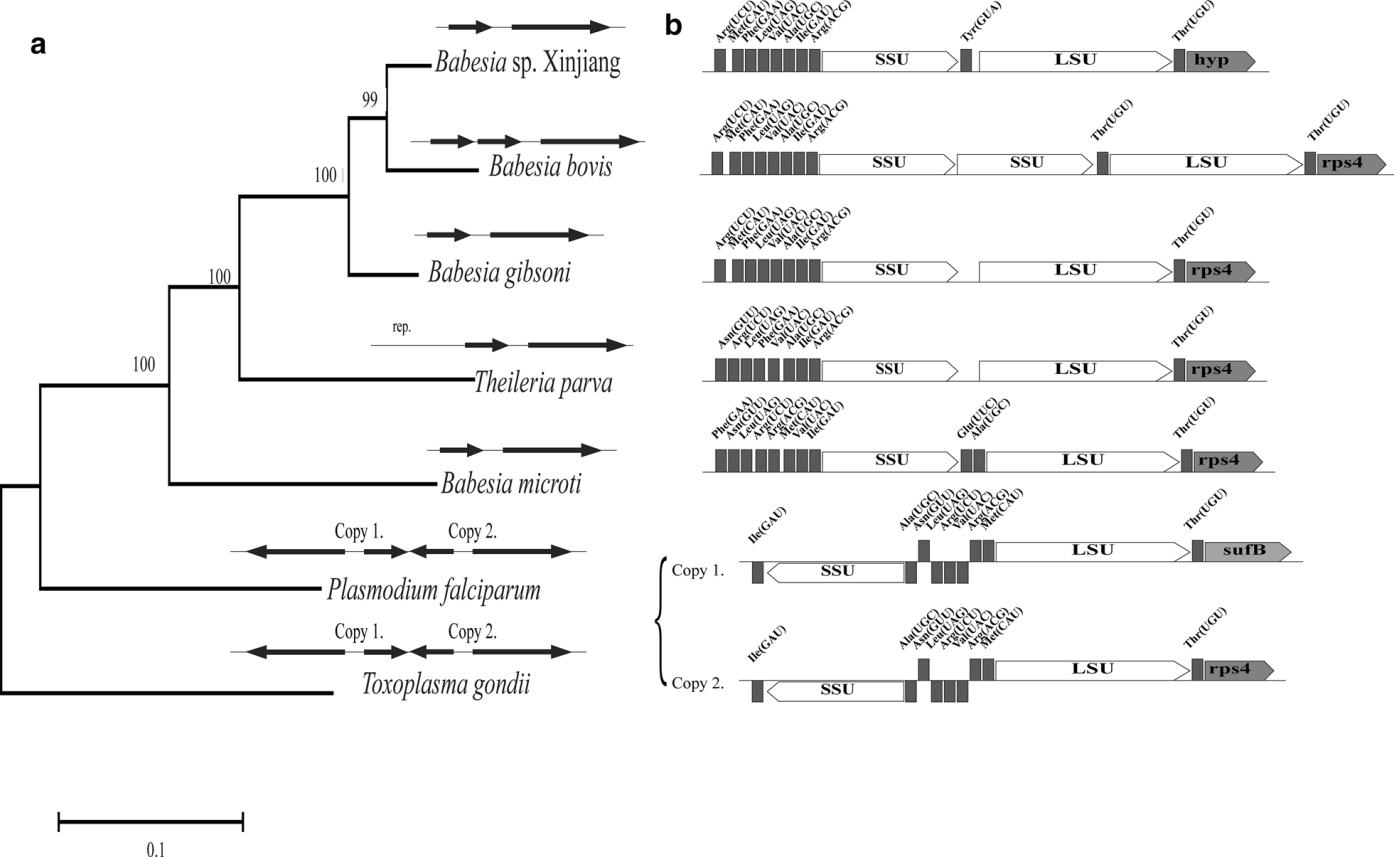
The evolutionary tree and genetic structure of rDNA region in the apicoplast genomes of *Babesia gibsoni* and other apicomplexan parasites. **a** Phylogenetic analysis based on the rDNA genes (*LSU* gene and *SSU* gene). The maximum likelihood method is used to construct the phylogenetic tree of rDNA genes (bootstrap > 99%), and the other two methods are used for correction. A simple schematic of the rDNA genes region in the apicoplast genome is shown at the top of each branch. **b** Gene structure map of rDNA genes region. The genetic structure of the rDNA region of *P. falciparum* and *T. gondii* is shown in the same figure due to their high similarity

Cluster 4 contains rDNA genes (*LSU* and *SSU*). The *SSU* and *LSU* genes are all single copies in *Babesia* spp. except for *B. bovis*, with the same transcription direction for all of them. However, the *SSU* and *LSU* are a set of multi-copy genes with opposite transcription direction in the apicoplast genomes of *T. gondii* and *P. falciparum* (Fig. 5). Meanwhile, the gene content and gene sequence in the cluster vary among species. In *P. falciparum* and *T. gondii*, the *SSU* and *LSU* genes are in the opposite direction with multiple copies; in *B. gibsoni* and *T. parva*, there is no tRNA between *SSU* and *LSU* genes; in *B. bovis*, there are two copies of the *SSU* gene and 2 Thr (UGU) tRNAs (Fig. 5). The formation of this genetic structure may be related to the occurrence of mutations during evolution of *B. bovis* [10, 14, 30, 38].

The genetic structure of each cluster and the region between clusters is different. Cluster 4 and Cluster 1 showed high similarity in regions, in contrast to the obvious difference between Cluster 1 and Cluster 2 (Fig. 4). In *Babesia* spp., the region between Cluster 1 and Cluster 2 is composed of multiple hypothetical protein regions, while in *B. microti*, the hypothetical protein was substituted by two tRNA genes for Gln (UUG) and Trp (CCA). Interestingly, the two coding regions described above are present in *T. parva*, along with two other tRNAs. These data suggest a high mutation rate in the region between Cluster 1 and Cluster 2 during evolution of the species, which can be speculated as a hotspot for recombination, and the region near Cluster 1 may have undergone significant recombination events during the evolution of Piroplasmida (Fig. 4). As mentioned above, the gene sequence of *rp*l11-*Clp*C is highly conserved in *T. gondii* and *Chromera*, but the *rp*l11 gene is absent in the apicoplast genomes of parasites within the Class Aconoidasida (which includes Piroplasmida and Haemosporida). Moreover, previous research has shown the occurrence of the rearrangement of the tRNA adjacent to the *Clp*C gene, so it was speculated that the deletion of the *rp*l11 gene might be related to the rearrangement of tRNA (Figs. 3, 4). The two stages, i.e. (i) from the free-living protoapicomplexan to the first apicomplexan, and (ii) from the ancestor of the piroplasm and coccidian to the first hematozoan, are shown to be important in the evolution of the parasite, due to the occurrence of gene deletion events and gene rearrangements in these two stages [38]. Therefore, we speculate that only a few simple genes were maintained in the apicoplast genome from these two stages of evolution, with some of them being transferred to the nuclear genome and then targeted to the apicoplast for function.

## Conclusions

In this study, we sequenced, assembled and annotated the apicoplast genome of *B. gibsoni.* The apicoplast genome of *B. gibsoni* is a 28.4 kb circular genome, with a high similarity in genetic structure and characteristics to those of other apicomplexan parasites (e.g. *Babesia* spp., *Plasmodium* spp., *Toxoplasma* spp.). *Babesia gibsoni* apicoplast genes have been gradually lost or transferred to the nuclear genome during evolution. Currently, we are trying to identify the proteins encoded by the nuclear genome and targeted to the apicoplast to improve our understanding of its metabolic pathways. This study provided a genetic basis for screening novel drug targets against babesiosis.

## Supplementary information

**Additional file 1: Table S1.** PCR primers used in sequencing the apicoplast genome of *Babesia gibsoni*. **Table S2.** Comparison of tRNA in the populations of *P. falciparum*, *T. gondii*, *B. microti*, *B. gibsoni* and *B. orientalis.*

**Additional file 2: Figure S1.** PCR results of amplified fragments of the apicoplast genome from *Babesia gibsoni* gDNA. Lane M: Marker; Lanes 1, 2: 35#; Lanes 3, 4: 37#; Lane 5: 38#; Lanes 6, 7: 31#; Lanes 8, 9: 34#; Lanes 10, 11: 32#; Lanes 12, 13: JTC-2; Lane 14, 15: JTC-3; Lane 16: *Tuf*A; Lanes 17, 18: 42#; Lanes 19, 20: 41#; Lanes 21, 22: 44#; Lanes 23, 24: 47#.

**Additional file 3: Figure S2.** Molecular phylogenetic analysis by maximum likelihood method. Evolutionary analyses were conducted in MEGA7. The genes analyzed by the evolutionary tree are present in all of these species (*rp*l4, *rpl*2, *rps*19, *rps*3, *rpl*16, *rpl*14, *rps*8, *rpl*6, *rps*5, *rpl*36, *rps*11, *rps*12, *rps*7, *Tuf*A).

**Additional file 4: Figure S3.** Domain structure of the *Clp*C chaperone of piroplasma. Two *Pfam*A domains were found in ClpC proteins of *Babesia* spp.: AAA_2 (ATPase catalytic function).

## Data Availability

Data supporting the conclusions of this article are included within the article and its additional files. The apicoplast genome sequence of *Babesia gibsoni* generated during this study was submitted to the NCBI GenBank database under the accession number MN481613. The datasets used and analyzed in this study can be obtained from the corresponding author upon reasonable request.

## Abbreviations

tufA: 1 EF-Tu elongation factor
tRNA: Transfer RNA
rRNA: Ribosomal RNA
WH: Wuhan strain
hp: Hypothetical protein
IPP: Isopentene pyrophosphate pathway
LSU: Large-subunit ribosomal
SSU: Small-subunit ribosomal
rpl: Ribosomal protein large subunit
rps: Ribosomal protein small subunit
hp: Hypothetical protein
