# Supplementary material for: Annotation and characterization of Babesia gibsoni apicoplast genome
Source: Parasit Vectors. 2020 Apr 21;13:209. doi: 10.1186/s13071-020-04065-7 (PMC7175588; doi:10.1186/s13071-020-04065-7)
Supplement: Supplementary file 1 — Additional file 1: Table S1. PCR primers used in sequencing the apicoplast genome of Babesia gibsoni. Table S2. Comparison of tRNA in the populations of P. falciparum, T. gondii, B. microti, B. gibsoni and B. orientalis. [file 13071_2020_4065_MOESM1_ESM.docx]

**Additional file 1**

**Table S1**

PCR primers used in sequencing the apicoplast genome of *Babesia gibsoni*.

| primer | primer sequence (5’→3’) | Position |
| --- | --- | --- |
| F34 | ACATATAATAGGATTATTAGATAG | 127-1662 |
| R34 | ATAATGACCTACCTATAGTAGT |  |
| F35 | TTTGAGTATATATCTATATTTCCT | 76-2663 |
| R35 | ATAATTACCATGTGTAGTAGC |  |
| JTC-F2 | ATAATTATACAACATTAATAGAATTAAAT | 3726-8256 |
| JTC-R2 | GCGGAATATTTAATGTATTAACTTT |  |
| JTC-F3 | GGTATACAGTCACATCAGAAT | 7698-13562 |
| JTC-R3 | TCTGCAATTATATATTTTAATTAAATTAT |  |
| F44 | CTTAGATACATAATAGGTAAGTATATC | 12441-14498 |
| R44 | GTACAGTTTTTATACCTTTACATTT |  |
| F31 | ATAAAGGTAAAATAACAGTTAG | 13830-15457 |
| R31 | ATACTAACCAACATTTTATACCT |  |
| F41 | AATTAATTTATTCTGGATGTTTAACT | 15293-21366 |
| R41 | CATATTCTACGTGTTTTGTATTAATAG |  |
| Tufa-F | ATGATAAAGGAACAATACATTAAAAAT | 21153-22373 |
| Tufa-R | ATCGATTATGATACCTGCACC |  |
| F37 | GAAGACATACAGCTTTTACTAT | 22135-24737 |
| R37 | CTTAATAAAGGATTAAATATAATTATATC |  |
| F32 | AAAAAACTAACAACATATTTATC | 24169-26473 |
| R32 | TTATTTAATTTAACTAAAGGTCT |  |
| F33 | AGAGTATAATACTATATATAAATCC | 27307-1-1760 |
| R33 | TGATTTTAATTCTAATAATAAACAT |  |
| F38 | TTACGAGTAATTTAGGTATAAC | 26173-1-2317 |
| R38 | TTAAACTATCATAACTAAATAATACTC |  |
| F42 | TTTATTAGCATACTTACATAATAAATTT | 28225-1-3194 |
| R42 | AATTAACTATAGGTCTTAAAGTATTT |  |
| F47 | CAATTAAATTTATTAGCATACTTACAT | 28217-1-3752 |
| R47 | AATGAAGATTCTCCTTTAATATTTAAT |  |

According to the principle of primer design, the sequences of conserved genes were amplified first, followed by amplifying sequences between two conserved genes until entire apicoplast genome is completely covered. After alignment with the raw sequence, the final genome size of the apicoplast of *Babesia gibsoni* was 28,386 bp. 31#: rpl2-rps3; 32#: ClpC1-ClpC2; 34#: RpoC1; 35#: RpoC2a-rps2; 37#: TufA-ClpC1-ClpC2; 38#: ClpC1-ClpC2-RpoC1; 42#: part of 38 #; 44#: JTC3-31#.

**Table S2**

Comparison of tRNA in the populations of *P. falciparum*, *T. gondii*, *B.microti*, *B. gibsoni* and *B. orientalis*.

| *P. falciparum* | | *T. gondii* | | *B.microti* | | *B. orientalis* | | *B. gibsoni* | |
| --- | --- | --- | --- | --- | --- | --- | --- | --- | --- |
| Ala | GCA | Ala | GCA | Ala | UGC | Ala | UGC | Ala | UGC |
| Ala | GCA | Ala | GCA |  |  |  |  |  |  |
| Arg | UGU | Arg | CGU | Arg | UCU | Arg | UCU | Arg | UCU |
| Arg | CGU | Arg | AGA | Arg | ACG | Arg | ACG | Arg | ACG |
| Arg | AGA | Arg | CGU |  |  |  |  |  |  |
| Arg | CGU | Arg | AGA |  |  |  |  |  |  |
| Asn | AAC | Asn | AAC | Asn | GUU | Asn | GUU | Asn | GUU |
| Asn | AAC | Asn | AAC |  |  |  |  |  |  |
| Asp | GAC | Asp | GAC | Asp | GUC | Asp | GUC | Asp | GUC |
| Cys | UGC | Cys | UGC | Cys | GCA | Cys | GCA | Cys | GCA |
| Gln | CAA | Gln | CAA | Gln | UUG | Gln | UUG | Gln | UUG |
| Glu | GAA | Glu | GAA | Glu | UUC | Glu | UUC | Glu | UUC |
|  |  |  |  | Glu | UUC |  |  |  |  |
| Gly | GGU | Gly | GGA | Gly | UCC | Gly | UCC | Gly | UCC |
| Gly | GGA |  |  |  |  |  |  |  |  |
| His | CAC | His | CAC | His | GUG | His | GUG | His | GUG |
| Ile | AUC | Ile | AUC | Ile | GAU | Ile | GAU | Ile | GAU |
| Ile | AUC | Ile | AUC |  |  |  |  |  |  |
| Leu | CUA | Leu | CUA | Leu | UAG | Leu | UAG | Leu | UAG |
| Leu | CUA | Leu | CUA |  |  |  |  |  |  |
| Lys | AAA | Lys | AAA | Lys | UUU | Lys | UUU | Lys | UUU |
| Met | AUG | Met | AUG | Met | CAU | Met | CAU | Met | CAU |
| Met | AUG | Met | AUG | Met | CAU | iMet | CAU | Met | CAU |
| Met | AUG | Met | AUG |  |  |  |  |  |  |
| Phe | UUC | Phe | UUC | Phe | GAA | Phe | GAA | Phe | GAA |
| Pro | CCA | Pro | CCA | Pro | UGG | Pro | UGG | Pro | UGG |
| Ser | AGC | Ser | AGC | Ser | UGA | Ser | GCU | Ser | GCU |
| Ser | UCA | Ser | UCA |  |  | Ser | UGA | Ser | UGA |
| Thr | ACA | Thr | ACA | Thr | UGU | Thr | UGU | Thr | UGU |
| Thr | ACA | Thr | ACA |  |  |  |  |  |  |
| Trp | UGG | Trp | UGG | Trp | CCA | Trp | CCA | Trp | CCA |
| Tyr | UAC | Tyr | UAC | Tyr | GUA | Tyr | GUA | Tyr | GUA |
| Val | GUA | Val | GUA | Val | UAC | Val | UAC | Val | UAC |
| Val | GUA | Val | GUA |  |  |  |  |  |  |
| (a-b) * | |  | UUA | iMet | UAU | Leu* | UAA | **？** | |
|  |  | Leu* |  |  |  |  |  |  |  |
|  |  |  |  |  |  |  |  |  |  |

* represent the tRNA containing the introns.

The same color represents the results of comparison.

**？**Not sure.
