# Supplementary figures and images for: Annotation and characterization of Babesia gibsoni apicoplast genome
Source: Parasit Vectors. 2020 Apr 21;13:209. doi: 10.1186/s13071-020-04065-7 (PMC7175588; doi:10.1186/s13071-020-04065-7)

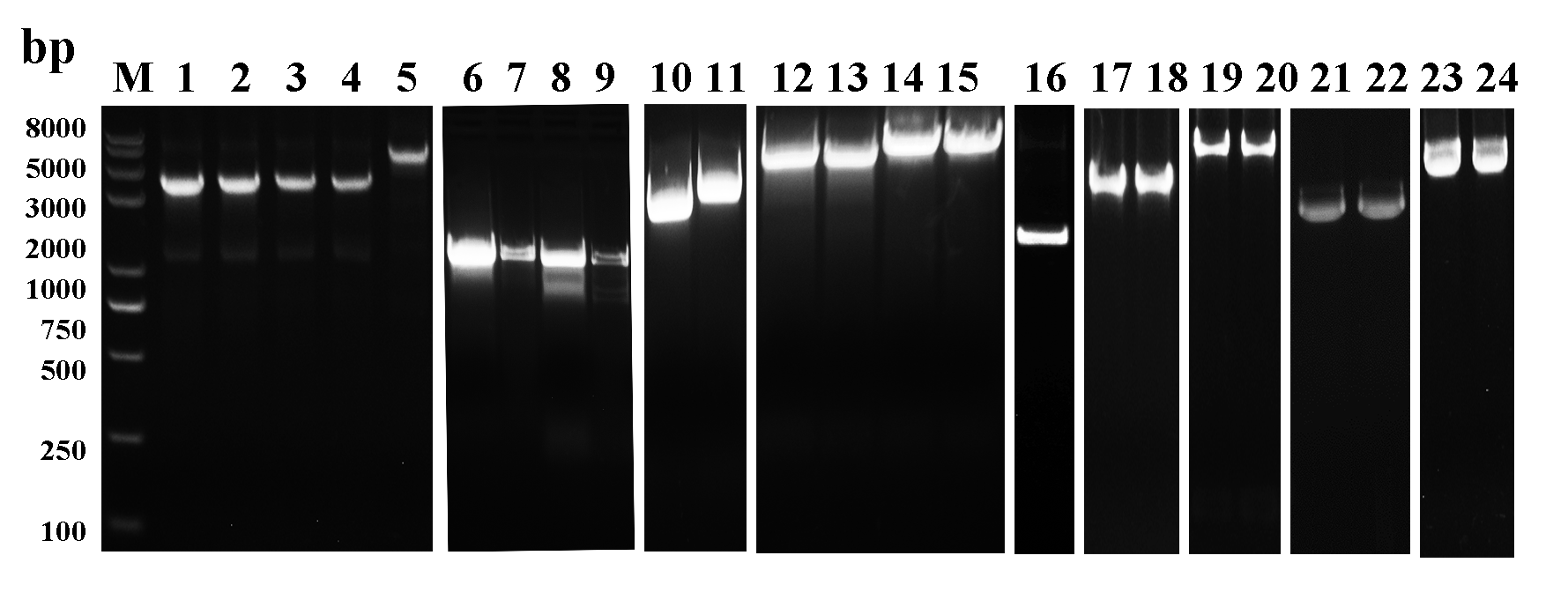

Supplement: Supplementary file 2 — Additional file 2: Figure S1. PCR results of amplified fragments of the apicoplast genome from Babesia gibsoni gDNA. Lane M: Marker; Lanes 1, 2: 35#; Lanes 3, 4: 37#; Lane 5: 38#; Lanes 6, 7: 31#; Lanes 8, 9: 34#; Lanes 10, 11: 32#; Lanes 12, 13: JTC-2; Lane 14, 15: JTC-3; Lane 16: TufA; Lanes 17, 18: 42#; Lanes 19, 20: 41#; Lanes 21, 22: 44#; Lanes 23, 24: 47#. [file 13071_2020_4065_MOESM2_ESM.tif]

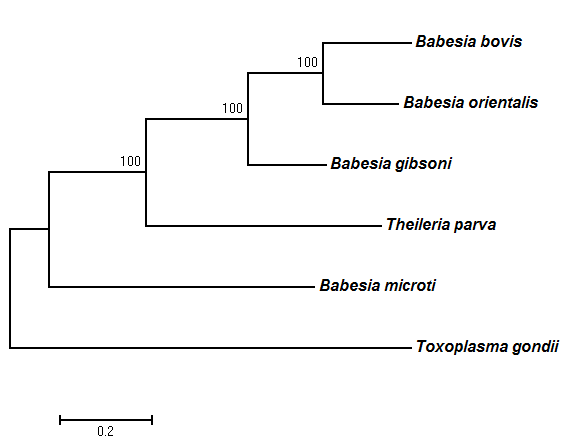

Supplement: Supplementary file 3 — Additional file 3: Figure S2. Molecular phylogenetic analysis by maximum likelihood method. Evolutionary analyses were conducted in MEGA7. The genes analyzed by the evolutionary tree are present in all of these species (rpl4, rpl2, rps19, rps3, rpl16, rpl14, rps8, rpl6, rps5, rpl36, rps11, rps12, rps7, TufA). [file 13071_2020_4065_MOESM3_ESM.tif]

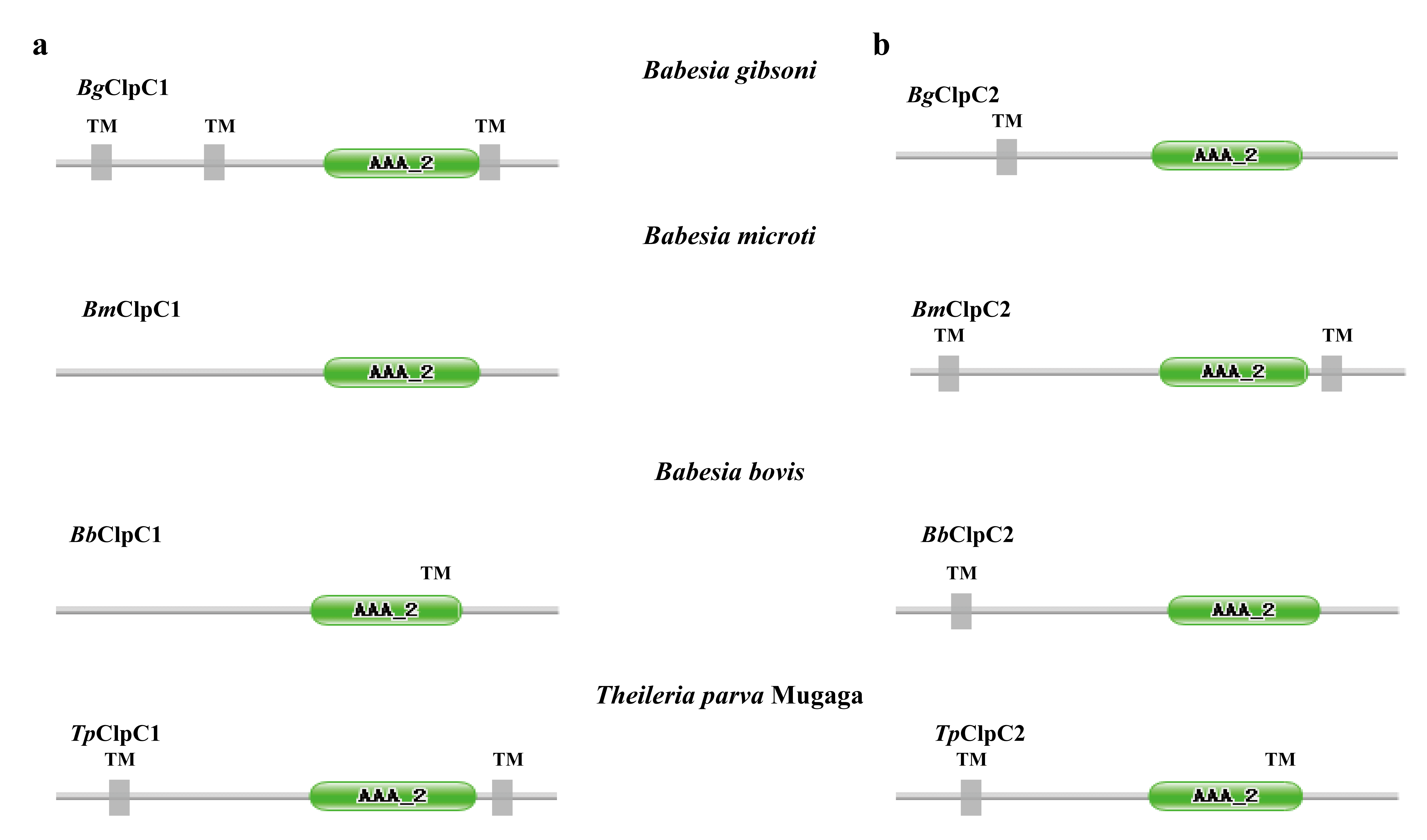

Supplement: Supplementary file 4 — Additional file 4: Figure S3. Domain structure of the ClpC chaperone of piroplasma. Two PfamA domains were found in ClpC proteins of Babesia spp.: AAA_2 (ATPase catalytic function). [file 13071_2020_4065_MOESM4_ESM.tif]
